# Affective Priming Using Facial Expressions Modulates Liking for Abstract Art

**DOI:** 10.1371/journal.pone.0080154

**Published:** 2013-11-19

**Authors:** Albert Flexas, Jaume Rosselló, Julia F. Christensen, Marcos Nadal, Antonio Olivera La Rosa, Enric Munar

**Affiliations:** 1 Human Evolution and Cognition Group, Associated group to IFISC (University of the Balearic Islands-CSIC), Palma de Mallorca, Balearic Islands, Spain; 2 Department of Psychology, University of the Balearic Islands, Palma de Mallorca, Balearic Islands, Spain; 3 Department of Basic Psychological Research and Research Methods, University of Vienna, Vienna, Austria; CSIC-Univ Miguel Hernandez, Spain

## Abstract

We examined the influence of affective priming on the appreciation of abstract artworks using an evaluative priming task. Facial primes (showing happiness, disgust or no emotion) were presented under brief (Stimulus Onset Asynchrony, SOA = 20ms) and extended (SOA = 300ms) conditions. Differences in aesthetic liking for abstract paintings depending on the emotion expressed in the preceding primes provided a measure of the priming effect. The results showed that, for the extended SOA, artworks were liked more when preceded by happiness primes and less when preceded by disgust primes. Facial expressions of happiness, though not of disgust, exerted similar effects in the brief SOA condition. Subjective measures and a forced-choice task revealed no evidence of prime awareness in the suboptimal condition. Our results are congruent with findings showing that the affective transfer elicited by priming biases evaluative judgments, extending previous research to the domain of aesthetic appreciation.

## Introduction

Aesthetic appreciation is ubiquitous. We frequently value the aesthetic qualities of our surroundings and other people in terms of beauty, liking, or attractiveness. Psychological aesthetics aims to understand how these aesthetic responses arise from the interaction of object, person and context features [1]. However, influenced by formalist art theories, which conceive aesthetic appreciation fundamentally as a response to relations among formal features such as lines, forms, colors or spaces, psychologists have mostly focused on determining how object properties (e.g., complexity, symmetry, and proportion) impact aesthetic appreciation. A different and broader approach conceives the spectator not as a passive receiver of form, but as an active agent that contributes to shape aesthetic experiences into personal and unique events. Knowledge about an object, for instance, has a profound impact on people’s appreciation of it [2–4]. But people bring much more to the aesthetic experience than acquired knowledge. They engage with the objects of aesthetic experience in a certain mood, or after encountering other affective objects. This is the case, for instance, with museum visits, where many different artworks are viewed in succession. The goal of the present paper is to ascertain the extent to which previous affective states and experiences influence aesthetic appreciation.

Experimental psychologists have demonstrated that, in many contexts, the affective qualities of stimuli can influence the subsequent evaluation of other stimuli or situations, even when the initial processing is irrelevant to the task or lacks subjective awareness [5,6]. Affective states and processes can exert a strong influence on cognitive operations and, particularly, on high-level cognition, including interpretation, judgment, decision-making, and reasoning [7].

As an explanation for these effects, it has been suggested that affect biases the encoding, retrieval, and processing of information used to formulate evaluations and judgments, especially when demanding and constructive processing is required [8]. From the field of implicit social cognition, the Affect Misattribution Procedure (AMP) has been devised in order to study how affect elicited by unaware primes influences subsequent evaluations [9]. The results of a number of studies using the AMP are congruent with the assumption that the affective response to suboptimal primes is transferred (unconsciously misattributed) to the evaluative judgment. Among the factors that modulate the effects of affective priming, the stimuli onset asynchrony (SOA) and the kind of stimulus used as prime have received most attention. Comparisons of the impact of different SOAs have increased our understanding of how evaluation is biased by emotional information processing at different levels of awareness [6,10]. It seems well established that SOAs under 300ms lead to the most robust affective priming effects [11]. However, studies using facial expressions as primes have reported conflicting results. For instance, it was found that when facial expressions were displayed with SOAs of 1000ms priming effects were weaker (or even non detectable) than when a SOA 5ms was used [6]. Such results suggest that the strength of the priming effects of facial expressions var with SOA duration, as other kinds of visual stimuli. Other studies, conversely, found strong priming effects both with brief (17ms) and extended (1000ms) SOAs [12]. The importance of this discrepancy resides in the fact that the SOA is a crucial moderator of affective priming effects, playing a determinant role in participants’ awareness of primes. In any case, a SOA of 30ms seems to be short enough to prevent awareness when backward masking is applied [13].

Various classes of prime stimuli seem to have the potential to transfer their affective valence to target stimuli, including facial expressions, as noted above [6,12]. Since emotionally loaded stimuli are processed thoroughly, and facial expressions of emotion are natural affective stimuli with a relevant evolutionary significance, it is not surprising that numerous experimental studies report that certain expressions are perceived faster and more accurately than others. Some results [14] suggest that negative facial expressions with a high adaptive value (e.g., fear) are more perceptually or emotionally salient than happiness or neutral expressions. In contrast, other studies, such as those using backward visual masking [15] lend support to the traditional notion of the happy face advantage.

This controversy has extended into the affective priming literature. Whereas some studies show that happy expressions are more easily and more readily processed, especially with brief SOAs [12,16,17], others show that negative stimuli, including facial expressions, elicit the fastest processing and the strongest response bias, even at different levels of perceptual awareness [18]. Because most of this work has focused on the emotional expressions of happiness, anger, sadness, and fear, little is known about the extent to which other kinds, such as expressions of disgust, have biasing effects on cognitive processes. Thus, despite recent interest in the emotion of disgust, sparked in part due to its distinctive underlying neural network, its affective priming effects, compared to those of other emotions, have hardly been investigated. Till our knowledge, only one recent study [19] contrasted subliminal processing of fear and disgust. Although noting that fear expressions lead to stronger subliminal priming effects than disgust, the results showed that expressions of disgust are capable of inducing priming effects, particularly when serving as alarms for social threat in a context of true interaction. In sum, there is a need to clearly ascertain whether facial expressions of happiness and disgust are capable of producing priming effects to the same extent, and whether there are differences depending on the SOAs (i.e., brief vs. extended). 

On the basis of the foregoing, the objective of this study is to ascertain the extent to which previously activated affective processing influences aesthetic liking for abstract art. As the above reviewed literature has shown, affective priming is an ideal paradigm to test the impact of affect on valuation. Given that affective responses are a critical source for people’s aesthetic appraisals [1], it can be assumed that the affective quality of briefly presented primes will influence the aesthetic evaluations of subsequently presented targets. The empirical study of the effects of affect misattribution on aesthetic appreciation has the potential of increasing our understanding of the effect of affective processes, and their temporal dynamics, on aesthetic evaluations [20–22]. Moreover, although the interaction between emotion and cognition has been suggested to be especially relevant in aesthetic evaluation tasks [1], very few experimental studies have directly examined how this interaction takes place.

In addition to extending the analysis of affect’s bias on evaluation into a new domain, the present study addresses the issues outlined above by means of a similar task to those used in evaluative priming research, incorporating some recent adaptations of the affect misattribution procedure [23]. 

Specifically, this study contrasts two main hypotheses. First, since it is presumed that the affect elicited by facial expression primes (happiness, disgust or no emotion) is transferred to the targets, differences in aesthetic evaluation of the target stimuli (abstract artworks) are expected. The direction of the effect is hypothesized to depend on the type of prime (lower ratings for disgust primes, higher ratings for happiness primes). Second, in line with previous research [6,16], the affective priming effect, both for disgust and happiness primes, is expected to be stronger in the suboptimal condition (brief SOA)—thus influencing the subsequent aesthetic appreciation to a greater degree—than in the optimal condition (extended SOA). Additionally, we designed the experiment to allow an exploration of the time course of the predicted priming effects over blocks.

## Methods

### Participants

Sixty-one undergraduate students, 30 female (mean age = 20.40, SD = 1.868) and 31 male (mean age = 20.23, SD = 1.586) volunteered to participate in this study. None of them had any kind of art expertise, and all had normal or corrected-to-normal vision. As an incentive, they received a breakfast voucher for their participation. All participants gave written informed consent. The experiment was approved by the Ethical Committee of the Comunitat Autònoma de les Illes Balears (Spain).

### Materials and stimuli

Twenty-four pictures of facial expressions selected from the Montreal Set of Facial Displays of Emotion (MSFDE) [24] were used as primes. They were selected pseudo-randomly from the Latino and Caucasian sets to include 8 instances of each of the chosen expressions (i.e., happiness, disgust, and neutral), and to represent male and female models equally in each of the three emotional categories.

The target stimuli were 8 different portions cropped from 3 abstract paintings by Hans Hartung and 8 different portions cropped from 3 abstract paintings by Jackson Pollock. These artworks were selected according to the following criteria: First, all paintings had to be abstract because of the required ambiguity and to avoid undesired memory effects. Second, since a number of very similar targets were needed, the fragments to be used had to be taken from paintings belonging to a single series by each artist.

### Procedure

Participants were told that they would have to evaluate a series of artworks in terms of how much they liked them aesthetically. They were requested to follow their initial impression, not to meditate their response for too long. Participants were also told that, just before the artworks, they might briefly see pictures of faces, which in some trials might hardly even be noticed, and that they should ignore them. The task was divided into two phases and three blocks, with 96 trials per block. The aim of the main phase, consisting of the first two blocks, was to measure the affective priming effects on aesthetic appreciation. The inclusion of the second block allowed us to examine the potential changes in the affective priming effect over time. In contrast, the second phase (third block) aimed to determine participants’ level of awareness of the primes.

The first block consisted of 48 trials under optimal (SOA 300ms) conditions and 48 under suboptimal (SOA 20ms) conditions. The 96 trials were presented pseudo-randomly, avoiding displaying the same primes (facial expressions of emotion) or targets (artworks) consecutively. For each SOA condition, each target was presented three times, one for each type of prime (happiness, disgust, or neutral). Each trial started with a 500ms presentation of a fixation cross in the center of the screen. At the offset of the fixation cross, the prime was presented (for 20ms or 300ms), and was immediately followed (ISI = 0ms) by a target picture, which also served as a backward mask. Once the target appeared, participants had to evaluate their degree of aesthetic liking for each particular target through a 5-point Likert scale (1 = dislike very much; 5 = like very much). Target stimuli remained on the screen until participants responded. 

The second block was identical to the first one. Once the second block finished, participants were asked to complete a self-report questionnaire to assess their phenomenological awareness of the primes. The questionnaire consisted of two questions, one of them referred to the perception of the prime, and the other one referred to the perception of the facial expression. The following example illustrates both questions: “Throughout the latter set of items, how many times have you been able to perceive the face/facial expression that appears briefly before each painting?” In this case, the Likert scale indicated was: 1 = None, 2 = Sometimes, 3 = Approximately half of the times, 4 = Most of the times, 5 = All of the times. 

In the third block only the brief SOA was used, and a forced choice task was included after each trial. This way, participants had to rate again every artwork and, inmediately after that, they had to identify the prime that they had just seen before that artwork. Thus, after the target evaluation, the prime was presented again together with a distractor, which was either the same actor expressing another emotion, or another actor expressing the same emotion. The specific task participants were requested to perform —to discriminate the facial expression or the identity of the face —was randomized across trials. No temporal restrictions were imposed, so the two images remained on the screen until participants made their choice by pressing the button on the left (“1” key of the keyboard) or on the right (“2” key). The side of the screen on which the test faces appeared was also randomized (i.e., left vs. right, respect to the central horizontal axis). Both tasks were performed on 48 trials. After this block, another self-report questionnaire asked the participants the level of confidence that they had in their responses with two questions, both illustrated in the following example: “With what level of confidence have you chosen between the two faces/facial expressions we presented after each painting?” The Likert scale was: 1 = No confidence, 2 = Little confidence, 3 = Neither much nor little confidence, 4 = Very confident, 5 = With total confidence.

Participants performed 4 practice trials before each block (two with each SOA), in which they evaluated abstract paintings by other artists. Data from the practice trials were not analyzed. In order to prevent participants’ fatigue, they took a 5-minute break between blocks.

## Results

Because ratings in the two first blocks had to be based on participants’ initial impression, and because we wished to exclude anticipated answers, responses with latencies over 2000ms or below 300ms were excluded prior to analysis (2.84% of all responses, 1.81% and 1.03% respectively). A 2 x 3 x 2 ANOVA was conducted, with SOA (20ms vs. 300ms), Type of Prime (happiness, disgust, or neutral) and Block (1st vs. 2nd) as factors, and ratings for aesthetic liking as dependent variable.

We found a main effect of Type of Prime, F(1.243, 74.59) = 16.168, MSE = .759, p < .001, ηp 2 = .212, and a significant interaction between SOA and Type of Prime, F(1.8,107.9) = 5.803, MSE = .099, p = .005, ηp 2 = .088. Within the extended SOA level aesthetic liking ratings for artworks were significantly higher when primed with happiness expressions than when primed with disgust expressions, F(1,60) = 20.255, MSE = .276, p < .001, ηp 2 = .252, and than when primed with neutral expressions, F(1,60) = 17.242, MSE = .172, p < .001, ηp 2 = .223. They were significantly lower when primed with disgust expressions than neutral ones, F(1,60) = 7.465, MSE = .056, p = .008, ηp 2 = .111. In contrast, within the brief SOA level, priming with happiness expressions also increased the liking ratings in comparison with the disgust expressions, F(1,60) = 10.664, MSE = .171, p = .002, ηp 2 = .151, and the neutral expressions, F(1,60) = 12.058, MSE = .115, p = .001, ηp 2 =.167, but liking for the artworks did not differ when primed with disgust or neutral expressions, F(1,60) = .600, MSE = .051, p = .442.

On the other hand, there were significant differences in aesthetic liking as a result of SOA only for the disgust prime, F(1,60) = 7.638, MSE = .063, p = .008, ηp 2 = .113. The aesthetic liking scores for targets preceded by primes expressing disgust were lower in the extended SOA condition than in the brief SOA condition.

With regards to the variations of the affective priming effect over time, although the SOA x Block interaction was not significant, F(1,60) = 3.742, MSE = .042, p = .058, ηp 2 = .059, the effect size reaches the usual standard value for a medium effect. Nevertheless, the simple effects analyses, taking into account the Type of Prime, show no significant effects between blocks, both for brief and extended SOAs. In addition, the comparison of the effects of the Type of Prime between SOAs for each block showed no differences in the Block 1, neither for disgust, F(1,60) = 1.761, MSE = .086, p = .190, nor for neutral primes, F(1,60) = .466, MSE = .068, p= .498. However, the difference for happiness primes showed a trend effect, F(1,60) = 2.983, MSE = .079, p = .089, ηp 2 = .047, suggesting a tendency in the aesthetic liking ratings to be greater for the optimal priming condition for this kind of prime. In the case of the Block 2, the results showed no differences for happiness, F(1,60) = .349, MSE = .091, p = .557, nor for neutral primes, F(1,60) = 1.272, MSE = .049, p = .264. Conversely, the difference for disgust primes was significant, F(1,60) = 8.915, MSE = .107, p = .004, ηp2 = .129, revealing that ratings were lower in the optimal priming condition. Inspection of the means suggests that this significant difference seems to be caused by the fact that the aesthetic ratings for artworks following disgust primes decrease over blocks, though only in the optimal priming condition. In fact, in the suboptimal priming condition they were slightly higher in the second block. In contrast, ratings for artworks primed with expressions of happiness decrease over blocks both for optimal and suboptimal conditions (see Table 1).

**Table 1:** Mean liking ratings for each SOA, type of prime and block.

*Block*		1				2				Total (*Both 1 and 2 blocks*)		
*Type of prime*		Happy	Disgust	Neutral		Happy	Disgust	Neutral		Happy	Disgust	Neutral
*SOA*	300ms	3.03*	2.59	2.68		2.91*	2.50*	2.63		2.97*	2.54*	2.66
	20ms	2.94*	2.66	2.72		2.88*	2.68^	2.68		2.91*	2.66^	2.69

Notes: * indicates that the mean is significantly different from the other Type of prime in that block, or in both blocks (Total column)

^ indicates that the mean is significantly different from the other SOA condition (upper cell).

Regarding the level of awareness, both the self-report results and the forced choice task suggest that, for the brief SOA, the primes were hardly ever consciously perceived. Specifically, the conducted analysis show that participants’ choices did not differ from chance when discriminating the identity, t(60) = -.156, p = .877, r = -.02, or the expression of the faces presented as primes, t(60) = 1.809, p = .075, r = .22.

## Discussion

The first of this study’s three main objectives was to ascertain whether the selected affective primes (disgust, happiness, and neutral) produced different effects on aesthetic liking for abstract artworks. Our analyses revealed that this was indeed the case. With the extended and brief SOAs, aesthetic liking scores were higher when the artworks were preceded by happiness primes than by either of the other two kinds. Additionally, aesthetic ratings for artworks primed with expressions of disgust were lower than when primed with neutral expressions, though only when using the extended SOA. 

Our results, thus, support the happy face advantage hypothesis [17], showing that it holds under optimal and suboptimal conditions, and extend it to an aesthetic appreciation task. Following previous results not related with the aesthetics domain [9], it could be inferred that the affective transfer from the primes to the abstract artworks occurred to a greater extent for the happiness than for the disgust facial expressions. We found no evidence suggesting that the stronger evaluative bias elicited by negative primes observed in some previous studies [18] carries over into the domain of aesthetic evaluation.

Our second objective was to investigate whether happiness and disgust primes caused stronger effects on the aesthetic evaluation ratings for the brief SOA (suboptimal priming in which no evidence of prime awareness was found) than for the extended SOA (optimal priming with prime awareness). The results confirmed that SOA magnitude indeed modulated priming effects. Contrary to our hypothesis, although we found no differences between suboptimal and optimal priming condition for happiness facial expressions, the negative effect of disgust was weaker in the suboptimal priming condition. In fact, expressions of disgust in this condition had no greater impact on liking for the artworks than neutral expressions. 

This evidence, on the one hand, questions previous work suggesting that facial expression primes exert little or no effect under high awareness conditions [6,25], but it is congruent with the results obtained by other authors [12,13,26]. The stronger effect of the expression of happiness, on the other hand, fits well with prior studies showing its greater effectiveness or “salience” in relation to other facial expressions [12,17]. The fact that the priming effect of facial expressions of disgust was limited to the optimal priming condition could be due to the expression of disgust having a lower evolutionary relevance than other negative expressions, such as anger or fear [12]. It could be also related with the absence of a context of potential threat, a factor that has been shown to be crucial for producing suboptimal affective priming effects by means of facial expressions of disgust [19]. Further research is needed to study the effect of additional negative facial expressions and other types of emotional primes or target stimuli (including artworks), as well as to contrast the effects of different prime exposures.

Our last objective was to ascertain the between-blocks time course of the effects. Although results related to the variation of the priming effects between blocks are not conclusive, they moderately suggest that the effectiveness of the affective transfer remains longer for expressions of disgust than for expressions of happiness in the extended SOA condition. The endurance of the effect of priming with expressions of disgust with extended SOAs seems to underscore the temporal robustness and resistance to repetition of the effects of optimal priming by means of disgust faces on aesthetic appreciation.

In sum, our results show that previously described priming effects on the evaluation of ambiguous stimuli extend to the evaluation of abstract art, and indicate that people’s prior affective state contributes significantly to the aesthetic appreciation of art. Positive affect increases liking for abstract art, while negative affect decreases it, especially when induced using longer exposure times, such as those that characterize a museum visit. Our results suggest that the affective state induced by one artwork could influence the aesthetic experience of the next, and thus challenge the view that emphasizes the role of object features in the aesthetic experience and relegates the contribution of personal experiences and states to a marginal role. Further studies should explore the effects of affective priming on other aesthetic assessments such as aesthetic preference, “beauty” evaluations and aesthetic quality judgments [20], enriching the theoretical discussions about the role of the emotions in the emergence of aesthetic experience [22].
